# Notes and Notices

**Published:** 1890-01

**Authors:** 


### NOTES AND NOTICES.

Gunpowder Stains.—The unsightly condition produced by gunpowder stains, it is said, can be removed by first painting the skin with a solution of biniodide of ammonium in an equal part of distilled water, and then with dilute hydrochloric acid. Fortunately the general practitioner is rarely called upon to treat such cases, but when he meets with them he should bear the above in mind.

For "Black Eye" there is nothing to compare, says the *N. Y. Medical Times*, with the tincture of a strong infusion of capsicum annuum mixed with an equal bulk of mucilage of gum arabic and with the addition of a few drops of glycerin. This should be painted all over the bruised surface with a camel's-hair pencil and allowed to dry on, a second or third coating being applied as soon as the first is dry. If done as soon as the injury is inflicted, this treatment will invariably prevent the blackening of the bruised tissue. The same remedy has no equal in rheumatic sore or stiff neck.

He was fond of Coffee.—Abd-el-Kader Anasari Djezeri Hanabali, son of Mohammed, thus expresses his opinion of this delicious beverage:

"O coffee! thou dispellest the cares of the great; thou bringest back those who wander from the paths of knowledge. Coffee is the beverage of the people of God, and the cordial of His servants who thirst for wisdom. When coffee is infused into the bowl, it exhales the odor of musk. The truth is not known except to the wise, who drink it from the foaming coffee-cup. God has deprived fools of coffee, who, with invincible obstinacy, condemn it as injurious. Coffee is our gold, and in the place of its libations we are in the enjoyment of the best and noblest society. It is even as innocent a drink as the purest milk, from which it is only distinguished by its color. Tarry with thy coffee in the place of its preparation and the good God will hover over thee."—*Table Talk.*

"The Prevention of Colds" was the subject of a recent paper read before the New York Homœopathic Medical Society. In it the author declared that colds can be prevented by developing the elasticity and vigor of the skin. The skin should be prepared to meet and resist atmospheric cold by systematic and regulated exposures to cold treatment, which is easiest applied in the bath. We should begin, he said, with such a temperature as is easily within the reactive powers already present, when the time of exposure is properly regulated, and increase the demand for reactive effort as the ability to respond becomes greater. It is by a similar system that we develop the muscles. A case in point was that of a Boston man whose lungs, after an attack of pneumonia, were thought to be too much affected to bear another Northern winter. After spending several winters in the South, to the neglect of his business, he was hardened sufficiently for a Northern winter by trunk and spine rubbings twice a day, washing off with water gradually reduced in two weeks' time from 90° F. to 70° F., and maintained at this temperature all winter.

A Good Subject for Surgery.—The native Egyptian is an extremely good subject for surgical operation. Clot Bey, the founder of modern medicine in Egypt, has it that "it requires as much surgery to kill one Egyptian as seven Europeans. In the native hospital the man whose thigh is amputated at two o'clock is sitting up and lively at six." Shock is almost entirely unknown and dread of an impending operation quite an exception. In explanation may be noted the resignation inculcated by their religion, the very small proportion of meat they eat and the total absence of alcohol from their diet and in general their regular, abstemious out-of-door life.—*Science.*

